# Phylogenetic Analysis of Mitogenomic Data Sets Resolves the Relationship of Seven *Macropostrongyloides* Species from Australian Macropodid and Vombatid Marsupials

**DOI:** 10.3390/pathogens9121042

**Published:** 2020-12-12

**Authors:** Tanapan Sukee, Anson V. Koehler, Ross Hall, Ian Beveridge, Robin B. Gasser, Abdul Jabbar

**Affiliations:** Department of Veterinary Biosciences, Melbourne Veterinary School, Faculty of Veterinary and Agricultural Sciences, The University of Melbourne, Melbourne 3030, Australia; anson.koehler@unimelb.edu.au (A.V.K.); rossh@unimelb.edu.au (R.H.); ibeve@unimelb.edu.au (I.B.); robinbg@unimelb.edu.au (R.B.G.)

**Keywords:** *Macropostrongyloides*, kangaroos, wallabies, wombats, mitochondrial DNA, phylogenetics, next-generation sequencing

## Abstract

Nematodes of the genus *Macropostrongyloides* inhabit the large intestines or stomachs of macropodid (kangaroos and wallabies) and vombatid (wombats) marsupials. This study established the relationships of seven species of *Macropostrongyloides* using mitochondrial (mt) protein amino acid sequence data sets. Phylogenetic analyses revealed that species of *Macropostrongyloides* (*M. lasiorhini*, *M. baylisi*, *M. yamagutii*, *M. spearei*, *M. mawsonae* and *M. woodi*) from the large intestines of their hosts formed a monophyletic assemblage with strong nodal support to the exclusion of *M. dissimilis* from the stomach of the swamp wallaby. Furthermore, the mitochondrial protein-coding genes provided greater insights into the diversity and phylogeny of the genus *Macropostrongyloides*; such data sets could potentially be used to elucidate the relationships among other parasitic nematodes of Australian marsupials.

## 1. Introduction

The genus *Macropostrongyloides* is the largest group of nematodes of the subfamily Phascolostrongylinae, occurring predominantly within the large intestines of macropodid (kangaroos and wallabies) and vombatid (wombats) marsupials [1]. Yamaguti [2] erected the genus *Macropostrongyloides* to distinguish one species, previously placed in the genus *Macropostrongylus* and now known as *Macropostrongyloides lasiorhini* from the colon of wombats. *Macropostrongyloides* was differentiated from other strongyloid genera by the presence of four prominent tooth-like projections which arise from the wall of the buccal capsule and protrude beyond the mouth opening [1]. Subsequently, other species found to possess four tooth-like structures within the buccal capsules were transferred to the genus *Macropostrongyloides*. This resulted in the transfer of *Macropostrongylus baylisi*, from a wide range of macropodid species [3], and *Cyclostrongylus dissimilis*, from the stomachs of swamp wallabies (*Wallabia bicolor*) to the genus *Macropostrongyloides* [4]. Subsequently, a fourth species, *Macropostrongyloides yamagutii*, characterised by the presence of denticles and teeth surrounding the mouth opening, was described from the large intestine of the western grey kangaroo (*Macropus fuliginosus*) [1].

Early morphological [1] and electrophoretic data [5] suggested that *Macropostrongyloides baylisi* was a species complex comprising genetically distinct populations in different hosts. These findings were supported by a recent study of the first and second internal transcribed spacers (ITS-1 and ITS-2, respectively) of nuclear ribosomal DNA, which identified four genotypes within *M. baylisi* [6]. Subsequently, these genotypes were found to be morphologically distinct and therefore described as three new species, namely, *M. mawsonae* from the eastern grey kangaroo (*Macropus giganteus*), *M. woodi* from the red kangaroo (*Osphranter rufus*), and *M. spearei* from the eastern wallaroo (*Osphranter robustus robustus*) and the euro (*Osphranter robustus erubescens*). Consequently, *M. baylisi* was re-described and its primary host was redefined as the northern wallaroo (*Osphranter robustus woodwardi*) [7]. Although the ITS-1 and ITS-2 sequence data provided genetic differentiation within and between species of *Macropostrongyloides*, the relationships of most species remain unresolved due to limited nodal support in the phylogenetic trees constructed [6].

The use of mitochondrial (mt) genome sequence data can be advantageous for investigating nematode systematics [8,9]. The amino acid sequences derived from mt protein-coding genes have been shown to provide strong nodal support in phylogenetic analyses of strongylid nematodes that could not be achieved using short ribosomal DNA sequences [10]. Previously, a study of the genera *Hypodontus* and *Macropicola*, intestinal parasites of macropodid marsupials belonging to the same subfamily as *Macropostrongyloides*, demonstrated that the amino acid sequence data could be used effectively to validate pre-existing phylogenetic hypotheses [11]. Therefore, in the present study, we utilised data sets from mitochondrial protein-coding genes to explore the genetic relationship(s) of seven representatives of the genus *Macropostrongyloides*, and discuss the implications of these findings in relation to their taxonomy, phylogeography and evolution.

## 2. Results

### 2.1. Characteristics of the mt Genomes

The mt genomes (~13.7 bp) representing the seven species of *Macropostrongyloides* encoded 12 protein genes, including cytochrome *c* oxidase subunits (*cox*1–3), nicotinamide dehydrogenase subunits (*nad*1–6, 4L), cytochrome *b* (*cob*) and adenosine triphosphatase subunit (*atp*6), two ribosomal subunits (large (*rrn*L) and small (*rrn*S)), 22 transfer RNA (tRNA) genes and two non-coding regions (Supplementary Figure S1; Table S1), consistent with strongylid nematodes studied to date [8]. Identical to the *H. macropi* reference genome (GenBank: NC023098), the lengths of the protein-coding genes from large to small were in order: *cox*1 *> nad*5 *> nad*4 *> cob > nad*1 *> nad*2 *> cox*3 *> cox*2 *> atp*6 *> nad*6 *> nad*3 *> nad*4L. The size of the gene annotations was comparable to those of *Hypodontus*. The nucleotide composition of mt genes was A+T-biased. For all seven *Macropostrongyloides* species, the *cox*1 gene was the largest (1572 nt, 524 amino acids) and *nad*4L (234 nt, 78 amino acids) was the smallest gene (Supplementary Table S2). The most common initiation codons were ATA and ATT. For all seven *Macropostrongyloides* species, 10 genes had complete termination codons (TAA and TAG) and two genes (*nad*5 of all species except *M. spearei* and *M. woodi* and *cox*3) had incomplete stop codons that contained only the T of the first codon position consistent with *H. macropi* (Supplementary Table S3). Overall, three of the most commonly used codons were phenylalanine (TTT; 12–12.8%), leucine (UAA, TTA; 7.1–10.4%) and isoleucine (ATT; 6.1–7.7%) (Supplementary Table S4). A summary of the raw data generated from the next-generation sequencing of mt genomes of *Macropostrongyloides* species is provided in Supplementary Table S5.

### 2.2. Comparative Analyses of mt Genomes of Macropostrongyloides Species

**Figure 1:**
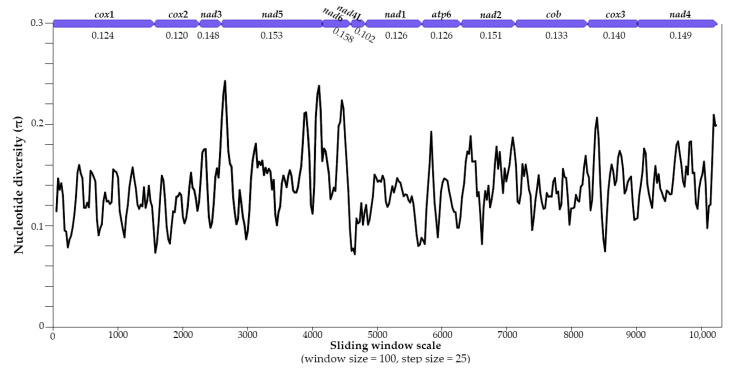
Sliding window analysis of concatenated protein coding mt gene sequences of *Macropostrongyloides* species. The black line indicates nucleotide diversity comparing all seven species of *Macropostrongyloides*. The average nucleotide diversity for each gene is indicated below the gene annotations (purple arrows). Nucleotide diversity measured iteratively every 25 bp over 100 bp windows of aligned mitochondrial DNA sequence data indicates peaks and troughs of sequence variability. Gene names and boundaries are indicated above the line plot.

The pairwise comparisons of nucleotide and amino acid sequences of the concatenated 12 protein-coding genes of *Macropostrongyloides* species are shown in Table 1. At the nucleotide level, sequence variation ranging from 11.6% (*M. woodi* versus *M. baylisi*) to 15.70% (*M. dissimilis* versus *M. baylisi*). A lower level of variation was detected in the amino acid sequences ranging from 4.28% (*M. spearei* versus *M. mawsonae*) to 8.74% (*M. dissimilis* versus *M. baylisi*). Pairwise comparison revealed that the sequences of *M. spearei* and *M. mawsonae* were the most similar at the amino acid level (4.28%), whereas *M. spearei* and *M. baylisi* were the most similar at the nucleotide sequence level (11.6%). *Macropostrongyloides dissimilis* and *M. baylisi* shared the least similarity in both amino acid (8.74%) and nucleotide (15.7%) sequences (Table 1). Pairwise comparisons of the amino acid sequence of each protein-coding gene revealed that *nad*2 and *nad*5 were the most variable (9.71–19.64% and 5.96–14.04%, respectively), whereas *cox*2 and *cox*1 were the least variable (0–3.9% and 0.38–3.9%, respectively). Sliding window analyses of the concatenated protein-coding genes revealed average nucleotide diversity values ranging from 0.102 (*nad*4L) to 0.158 (*nad*6), with it being higher across *nad6* (0.158), *nad*5 (1.53) and *nad*2 (1.51) genes (Figure 1).

### 2.3. Phylogenetic Analyses

**Figure 2:**
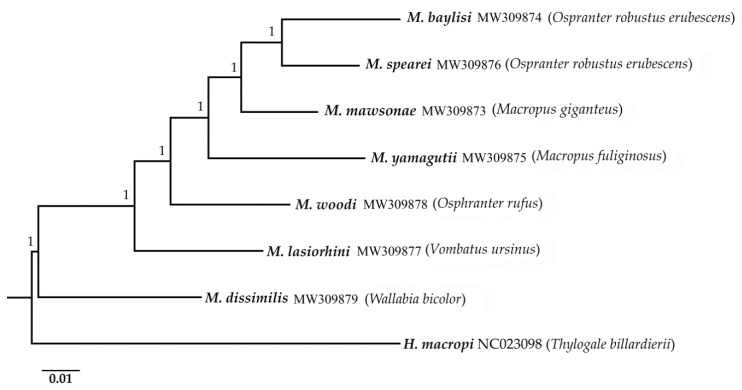
Phylogenetic analysis of the concatenated amino acid sequences of the mitochondrial protein-coding genes of seven species of *Macropostrongyloides* based on Bayesian inference analyses. The number above each tree branch indicates the statistical support based on the posterior probability score. *Hypodontus macropi* (GenBank: KF361318) was included as the outgroup. The scale bar indicates the number of inferred substitutions per amino acid site.

The Bayesian inference analyses revealed that *M. dissimilis* from the stomach of the swamp wallaby (*W. bicolor*) was the sister species to the remaining congeners, all of which inhabit the large intestine of their hosts (posterior probability [pp] = 1.00). *Macropostrongyloides lasiorhini* from the wombat was the sister species to the remaining species from kangaroos (pp = 1.00). Among the kangaroo-inhabiting species, *M. baylisi* and *M. spearei* from euros/wallaroos (*O. robustus*) were the most closely related species (pp = 1.00) (Figure 2).

## 3. Discussion

**Figure 3:**
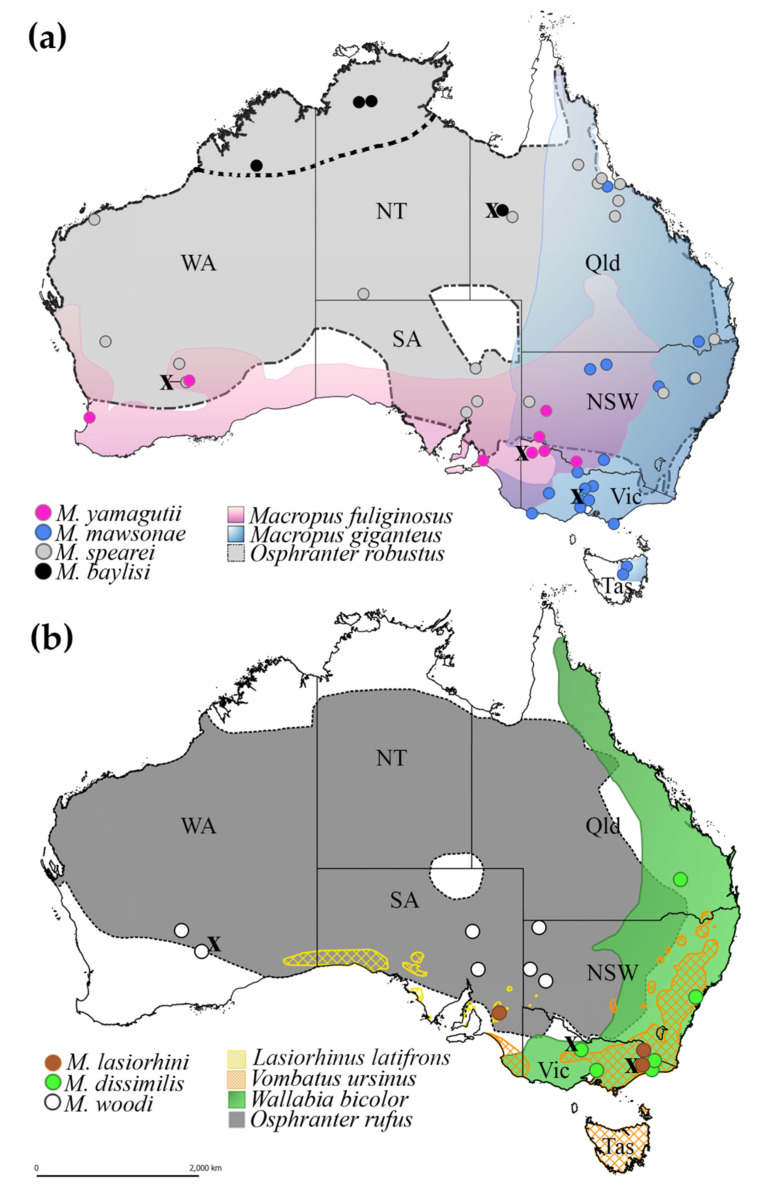
Distribution of the seven species of *Macropostrongyloides* in Australia. The localities in which specimens have been collected for amino acid sequences derived from mt protein-coding genes in this study are represented by an X. (**a**) The distribution of *Macropostrongyloides yamagutii*, *M. mawsonae*, *M. spearei* and *M. baylisi.* The dotted line in figure (**a**) indicates the distribution of the Northern wallaroos (*Osphranter robustus woodwardi*) limited to the northern parts of Western Australia and the Northern Territory. (**b**) The distribution of *M. lasiorhini*, *M. dissimilis* and *M. woodi.* Abbreviations: NSW = New South Wales, NT = Northern Territory, Qld = Queensland, SA = South Australia, Tas = Tasmania, WA = Western Australia, Vic = Victoria.

The phylogenetic tree inferred from the mt amino acid sequence data revealed that *M. baylisi* and *M. spearei* were closely related. However, the close association between these two species was not detected in the study based on ITS-1 and ITS-2 sequences [6]. The genetic similarly between *M. baylisi* and *M. spearei* correlates with their hosts, which are the same species of wallaroo (*Osphranter robustus*) but different sub-species. *Macropostrongyloides spearei* is found throughout the distribution of the eastern wallaroo (*O. r. robustus*) along the east coast of Australia and the euro (*O. r. erubescens*), which has a geographic distribution extending almost across the entire Australian mainland (Figure 3A). *Macropostrongyloides baylisi* primarily occurs in the northern wallaroo (*O. r. woodwardi*), distributed in the northern parts of the Northern Territory and Western Australia (Figure 3A); however, no specimen was available herein from this sub-species of host for DNA extraction. The specimen of *M. baylisi* included in this study was from a euro located at Cloncurry, Queensland. Prevalence data show that both *M. baylisi* and *M. spearei* are capable of infecting other host species which occur in sympatry with their primary hosts [6,7]. Co-evolution may have played a role in the speciation of *M. baylisi* and *M. spearei* in the two host sub-species. However, since there are no clear geographic barriers between the distribution of northern wallaroo and the euro, the species pair may have arisen from a host-switch, a mechanism that has been observed in the many of the strongyloid nematodes infecting Australian macropodid marsupials [12].

The phylogenetic tree inferred from the mt amino acid sequences also found a close association between *M. mawsonae* from the eastern grey kangaroo (*Macropus giganteus*) and *M. yamagutii* from the western grey kangaroo (*M. fuliginosus*), consistent with the findings of a previous study on *M. baylisi* using allozyme electrophoresis [5]. As with *M. baylisi* and *M. spearei*, the relationship between *M. mawsonae* and *M. yamagutii* is related to the distribution of their hosts. The eastern and western grey kangaroos are closely related species that evolved during the middle to late Pleistocene in the southeastern and southwestern parts of Australia, respectively [13]. The former species occupies grassland and open forests in high rainfall zone whereas the latter species occurs predominantly in semi-arid zones. However, the habitat of these two macropodid hosts overlaps in western Victoria, allowing their parasites to transfer between the hosts [14]. *Macropostrongyloides yamagutii* occurs commonly throughout the distribution of the western grey kangaroo. However, the distribution of *M. mawsonae* is concentrated in Victoria and southern New South Wales and is rarely encountered from eastern grey kangaroos in Queensland [6,7].

Phylogenetic analyses of the mt amino acid sequences show that *M. lasiorhini* from the common wombat (*Vombatus ursinus*) formed a sister species relationship to *Macropostrongyloides* from the large intestines of macropodid hosts. *Macropostrongyloides* is currently the only strongyloid genus with species occurring in both macropodid and vombatid marsupials. The position of *M. lasiorhini* in the phylogenetic tree was close to *M. woodi* from *O. rufus.* Current records show that *M. lasiorhini* occurs in the common wombat and the southern hairy-nosed wombat (*Lasiorhinus latifrons*) [1]. However, the geographic distribution of these hosts do not overlap (Figure 3B) and a previous molecular study (ITS-1 and ITS-2 data) suggests that *M. lasiorhini* from these two wombat hosts are genetically distinct [6].

The distant relationship between *M. dissimilis* and its congeners is strongly supported by the current mt and previous nuclear ITS-1 and ITS-2 data [6]. In addition to being the only species in the genus that occurs in the stomach of the host, *M. dissimilis* possesses some distinctive morphological features indicating that this species may have been incorrectly assigned to the genus *Macropostrongyloides*; therefore, future taxonomic revision is required. The predilection site within the stomach of *M. dissimilis* is unique not only to the genus *Macropostrongyloides* but also the subfamily Phascolostrongylinae. Two other species within the Phascolostrongylinae that inhabit the stomach of their hosts are *Paramacropostrongylus iugalis* and *Pamacropostrongylus typicus* from grey kangaroos [1]. The majority of strongyloid nematodes of macropodid marsupials that occur in the stomach of their hosts are grouped in the subfamily Cloacininae [15]. An unpublished study of the ITS sequence data found that *M. dissimilis*, *P. typicus* and *P. iugalis* were closely related, suggesting that the relationship among these taxa is influenced by the predilection sites within their hosts [16]. The predilection sites within the hosts have been found to be a key influence in the evolution within the nematode order Strongylida [17]. Furthermore, the anatomy of the gastrointestinal tract of the host is one of the key drivers of speciation in strongyloid nematodes of Australian marsupials [18]. The intestinal-dwelling species (i.e., subfamily Phascolostrongylinae) were hypothesised to be the earlier strongyloids to parasitise macropods. Subsequently, the evolution of large complex forestomachs in the Macropodidae led to the extensive radiation of the subfamily Cloacininae. Stomach-inhabiting phascolostrongyline species such as *M. dissimilis*, *P. iugalis* and *P. typicus* exhibit both apomorphic and plesiomorphic morphological characters, suggesting that these species are a link in the evolution of the Phascolostrongylinae and the Cloacininae [18]. However, this hypothesis requires further testing. Future studies on the mt sequences of *P. iugalis* and *P. typicus* in relation to *M. dissimilis* and the cloacinine species may provide greater insights into their evolution.

The sliding window analyses identified genes within the mt genomes of *Macropostrongyloides* that exhibited higher levels of nucleotide diversity than others, including *nad*2, *nad*5 and *nad*6, consistent with the mt genomes of *Hypodontus* and *Macropicola* [11]. Such regions could be targeted as potential molecular markers for future phylogenetic studies instead of sequencing the entire mt genome, which can be costly when examining a large number of samples. Although the present study yielded strong support (at the amino acid level) for the relationships within the genus *Macropostrongyloides*, data sets were obtained for a single specimen for each of the seven species. Future work might need to critically assess whether nucleotide variability in protein genes within individual species of *Macropostrongyloides* alters encoded amino acid sequences; the present hypothesis is that most, if not all, nucleotide alterations will be ‘silent’ (synonymous).

In conclusion, analyses of amino acid sequences derived from mt protein-coding genes provided greater insights into the relationships within the genus *Macropostrongyloides*. However, not all species of *Macropostrongyloides* could be included in the current study due to the unavailability of specimens suitable for molecular analyses. The species not included were *M. dendrolagi* from the tree kangaroo, *Dendrolagus dorianus* [19], and *M. eppingensis* from the northern hairy-nosed wombat, *Lasiorhinus kreftti* [20]. Future studies utilising mt amino acid sequence data could be very useful for understanding the phylogenetic relationships among strongyloid nematodes of Australian marsupials that have hitherto been heavily reliant on ITS sequence data.

## 4. Materials and Methods

### 4.1. Sample Collection and Morphological Identification

Adult specimens of *M. baylisi*, *M. spearei*, *M. mawsonae*, *M. woodi*, *M. yamagutii*, *M. lasiorhini* and *M. dissimilis* were acquired from the frozen parasite collection at the Melbourne Veterinary School, The University of Melbourne. The nematodes were collected from road-killed or culled macropodid and vombatid hosts (cf. [21]) from various localities across Australia (Table 2). The nematodes were stored at −80 °C or in 70% ethanol until required for DNA extraction. Individual nematodes were thawed and cut into three segments. The anterior and posterior extremities of each specimen were cleared in lactophenol for morphological identification and retained as voucher specimens, which were deposited in the South Australian Museum, Adelaide (SAM49067-49073). The mid-section of each nematode was washed three times in saline prior to DNA extraction, and the ITS-1 and ITS-2 sequences were defined for each specimen using an established method [6].

### 4.2. DNA Isolation and Sequencing of a Full Complement of mt Protein-Coding Genes

Total genomic DNAs were isolated from individual nematodes using the QIAamp DNA Micro Kit (Qiagen, Hilden, Germany) according to the manufacturer’s protocol. The quality and quantity of each DNA sample were determined using the 2200 TapeStation (Agilent, Santa Clara, CA, USA). TruSeq DNA libraries were prepared as per the manufacturer’s recommendations (Illumina, San Diego, CA, USA) and included: (i) end-repair and A-tailing of the 3′ ends, (ii) ligation of the adaptors, (iii) enrichment of the libraries and purification of the enriched library using Ampure Beads (Beckman Coulter, Brea, CA, USA). The libraries were assessed for quality using the 2200 TapeStation, pooled and sequenced on the Illumina MiSeq platform using the 300 cycle v3 reagent kit (2 × 150 paired-end reads).

For each nematode, raw DNA sequence data in the FASTQ format [22] were filtered for quality (Phred cut-off: 30) and trimmed using the program trimmomatic v.0.38 [23]. Individual mt protein-coding gene sequences were *de novo* assembled from at least 0.55 million filtered reads (50–150 bp) using the program Spades v. 3.13.0 [24] (employing default parameters) and annotated employing an established pipeline [10]. In brief, each protein-coding mt gene was identified by local alignment comparison (six reading frames) using amino acid sequences inferred from corresponding genes in the mt genome of a reference species *H. macropi* (GenBank accession no. NC0230998 [11]).

Sequence regions that were ambiguously aligned with the reference genome were verified by PCR amplification (specific primers were designed in Primer3) [25] and Sanger sequencing. For each sample, the nucleotide sequences were deposited in the GenBank database under BioProject no. PRJNA679672 and accession nos. MW309873–MW309879.

### 4.3. Sequence Comparisons and Sliding Window Analysis

The mt DNA sequences were aligned with published sequences of *H. macropi* using MUSCLE [26] and CLUSTAL W [27] in the MEGA software [28] followed by manual adjustments. Each of the 12 protein-coding genes was conceptually translated to amino acid sequences using the Invertebrate Mitochondrial Code. Nucleotide and amino acid sequence differences were calculated separately by pairwise comparison. Nucleotide diversity of aligned, concatenated sequences was determined by sliding window analysis (100 bp window, 25 bp step) using DnaSP v.6 [29]. The arrow icons indicating the genes boundaries were generated using the Geneious Prime 2020.2.4 software (http://www.geneious.com/) and overlaid on to the plot (Figure 1). Nucleotide diversity was plotted against the midpoint positions of each window, with the average nucleotide diversity calculated for each protein-coding gene.

### 4.4. Phylogenetic Analyses

For the phylogenetic analyses, amino acid sequences of the 12 protein-coding genes of *Macropostrongyloides* species and *H. macropi* (GenBank NC0230998) were concatenated and aligned (Mendeley Data doi: 10.17632/nnnm5cgcf3.1). The most suitable substitution model and partitioning schemes for the alignment were determined using PartitionFinder for amino acids [30]. The boundaries of each gene were specified, model selection was set to the Akaike information criterion and the greedy algorithm was used with branch lengths estimated as “unlinked” to search for the best-fit scheme. Bayesian inference (BI) analysis was performed in MrBayes v.3.2.7 [31]. Based on the best partitioning scheme determined by Partition Finder, the alignment was divided into seven subsets comprising Subset 1 (*cox*1), Subset 2 (*cox*2, *cox* 3, *nad*4L), Subset 3 (*nad*3, *cob*, *atp*6), Subset 4 (*nad*5, *nad* 6) Subset 5 (*nad*1), Subset 6 (*nad*1) and Subset 7 (*nad*4). The analysis was conducted using the Markov Chain Monte Carlo with four simultaneous tree building chains (three heated and one cold). Posterior probability was calculated for two million generations and sampled every 1000 generations. The standard deviation of split frequencies was <0.01 and the Potential Scale Reduction Factor value approached one. The consensus tree was generated from the final 75% of trees produced. The condensed tree was visualised in FigTree v. 1.4.2 [32].

## Figures and Tables

**Table 1:** Pairwise differences (%) in nucleotide (top) and amino acid (bottom) sequences of the 12 concatenated mt protein-coding genes of seven *Macropostrongyloides* species.

	*M. baylisi*	*M. mawsonae*	*M. spearei*	*M. woodi*	*M. dissimilis*	*M. lasiorhini*	*M. yamagutii*
*M. baylisi*	-	12.1	11.4	13.7	15.7	14.0	13.7
*M. mawsonae*	5.2	-	11.8	13.2	14.7	13.3	12.6
*M. spearei*	4.5	4.3	-	13.7	15.4	13.8	13.4
*M. woodi*	6.3	5.7	5.8	-	15.3	13.5	14.2
*M. dissimilis*	8.7	7.9	8.5	7.9	-	14.6	15.5
*M. lasiorhini*	7.1	6.3	6.5	5.8	7.6	-	13.9
*M. yamagutii*	6.7	5.7	6.4	6.3	8.9	7.0	-

**Table 2:** Details of specimens of the seven *Macropostrongyloides* species included in this study.

Nematode	Host Species (Scientific Name)	Predilection Site	Collection Locality	Coordinates	Voucher No.	GenBank Accession No.
*M. mawsonae*	Eastern grey kangaroo (*Macropus giganteus)*	colon	Heathcote, Vic	36°92′ S, 144°43′ E	W449	MW309873
*M. baylisi*	Euro (*Osphranter robustus erubescens)*	colon	70 km west of Cloncurry, Qld	20°46′ S, 139°53′ E	21V1	MW309874
*M. yamagutii*	Western grey kangaroo (*Macropus fuliginosus)*	colon	Hattah Lakes National Park, Vic	34°45′ S, 142°20′ E	DD4	MW309875
*M. woodi*	Red kangaroo (*Osphranter rufus)*	colon	Kalgoorlie, WA	30°44′ S, 121°28′ E	23Q1	MW309876
*M. lasiorhini*	Common wombat (*Vombatus ursinus)*	colon	Gippsland, Vic	37°30′ S, 147°51′ E	41R1	MW309877
*M. spearei*	Euro (*Osphranter robustus erubescens)*	colon	Kalgoorlie, WA	30°44′ S, 121°28′ E	23M1	MW309878
*M. dissimilis*	Swamp wallaby (*Wallabia bicolor)*	stomach	Kamarooka, Vic	36°28′ S, 144°22′ E	10W9	MW309879

Abbreviations: Qld = Queensland, Vic = Victoria, WA = Western Australia.

## Supplementary Materials

The following are available online at https://www.mdpi.com/2076-0817/9/12/1042/s1, Table S1: The nucleotide positions of the genes within the mitochondrial genomes of the seven *Macropostrongyloides* species included in the study; Table S2: The lengths and nucleotide composition of the entire mt genome, protein-coding genes (PCG), RNA genes and tRNA genes of *Macropostrongyloides* spp.; Table S3: The amino acid (aa) sequence lengths, initiation (ini.), and termination (ter.) codons of the mt protein-coding genes (PCG) of *Macropostrongyloides* spp.; Table S4: Codon usage in the 12 protein-coding genes of *Macropostrongyloides* spp. shown as the number of occurrences of each codon followed by percentage of the total usage in parentheses; Table S5: Summary of the raw data generated from the next-generation sequencing of mitochondrial genomes of *Macropostrongyloides* spp.; Figure S1: Schematic representation of the mitochondrial genome of *Macropostrongyloides* represented by the genome of *M. baylisi*. The protein coding sequences (CDS) are shown in blue, the transfer RNAs (tRNA) are shown in red, and the large (rrnL) and small (rrnS) ribosomal RNAs are shown in pink.
