# Supplementary material for: Phylogenetic Analysis of Mitogenomic Data Sets Resolves the Relationship of Seven Macropostrongyloides Species from Australian Macropodid and Vombatid Marsupials
Source: Pathogens. 2020 Dec 12;9(12):1042. doi: 10.3390/pathogens9121042 (PMC7763074; doi:10.3390/pathogens9121042)
Supplement: Supplementary file 1 [file pathogens-09-01042-s001.pdf]

Supplementary materials

Table S1. The nucleotide positions of the genes within the mitochondrial genomes of the seven *Macropostrongyloides* species included in the study.

Gene	Species						
	M. baylisi	*M. spearei*	*M. mawsonae*	*M. woodi*	*M. yamagutii*	*M. dissimilis*	*M. lasiorhini*
*cox*1	1–1572	1–1572	1–1572	1–1572	1–1572	1–1572	1–1572
*trn*C	1575–1629	1574–1630	1575–1633	1576–1635	1573–1628	1572–1629	1572–1628
*trn*M	1631–1692	1630–1692	1633–1694	1635–1695	1629–1690	1628–1688	1628–1688
*trn*D	1710–1767	1713–1769	1707–1765	1713–1772	1705–1763	1689–1745	1706–1764
*trn*G	1769–1826	1770–1825	1766–1823	1773–1830	1764–1820	1748–1805	1764–1820
*cox*2	1826–2521	1826–2521	1823–2518	1830–2525	1820–2515	1805–2501	1820–2515
*trn*H	2523–2581	2524–2582	2519–2578	2527–2583	2515–2573	2501–2558	2515–2573
*rrn*L	2589–3549	2592–3547	2586–3543	2590–3680	2582–3539	2563–3518	2577–3535
*nad*3	3551–3886	3549–3884	3545–3880	3682–4017	3541–3876	3520–3855	3537–3872
*nad*5	3988–5548	3988–5550	3983–5543	4124–5686	3974–5534	3960–5520	3965–5525
*trn*A	5544–5599	5550–5604	5544–5599	5686–5742	5535–5589	5521–5579	5526–5582
*trn*P	5848–5906	5844–5901	5839–5899	5979–6036	5835–5892	5832–5887	5814–5871
*trn*V	5913–5970	5909–5965	5900–5955	6049–6105	5905–5962	5890–5946	5887–5943
*nad*6	5970–6404	5966–6400	5955–6389	6105–6539	5962–6396	5946–6380	5943–6377
*nad*4L	6406–6639	6402–6635	6391–6624	6545–6778	6398–6631	6383–6617	6380–6613
*trn*W	6641–6698	6637–6695	6625–6682	6781–6839	6632–6691	6619–6676	6616–6674
*trn*E	6700–6756	6697–6753	6682–6737	6841–6897	6699–6755	6687–6744	6680–6736
*rrn*S	6757–7454	6754–7449	6738–7432	6898–7595	6756–7452	6745–7439	6737–7433
*trn*S (UCN)	7455–7510	7450–7506	7433–7487	7596–7650	7453–7506	7440–7495	7434–7489
*trn*N	7510–7566	7506–7562	7486–7544	7650–7706	7506–7562	7494–7552	7489–7543
*trn*Y	7568–7621	7562–7618	7551–7607	7712–7768	7572–7629	7557–7613	7549–7604
*nad*1	7621–8493	7618–8490	7606–8478	7768–8640	7626–8502	7613–8485	7601–8477
*atp*6	8503–9102	8499–9098	8486–9085	8656–9255	8508–9107	8493–9093	8481–9081
*trn*K	9104–9167	9100–9164	9087–9148	9258–9320	9109–9172	9097–9160	9084–9146
*trn*L (UUR)	9172–9227	9169–9224	9156–9212	9323–9378	9174–9230	9182–9237	9148–9203
*trn*S **(AGN)**	9227–9281	9224–9277	9211–9266	9378–9432	9229–9285	9218–9291	9261–9334
*nad*2	9286–10125	9280–10122	9268–10110	9440–10276	9287–10129	9293–10135	9339–10178

*trn*I	10129–10186	10126–10182	10114–10172	10279–10334	10132–10191	10142–10198	10179–10237
*trn*R	10187–10243	10182–10237	10181–10236	10339–10395	10190–10246	10199–10253	10242–10296
*trn*Q	10249–10304	10242–10297	10238–10293	10404–10459	10246–10301	10261–10316	10298–10353
*trn*F	10316–10372	10313–10369	10312–10368	10478–10535	10323–10379	10323–10382	10359–10416
cob	10372–11484	10369–11481	10368–11480	10535–11647	10380–11492	10381–11493	10416–11528
*trn*L (CUN)	11484–11539	11480–11537	11480–11535	11647–11702	11492–11550	11493–11548	11528–11582
*cox*3	11539–12303	11537–12301	11535–12300	11702–12467	11550–12315	11548–12313	11582–12347
*trn*T	12305–12362	12303–12360	12301–12357	12468–12526	12316–12372	12314–12372	12348–12402
*nad*4	12363–13592	12360–13589	12357–13586	12526–13755	12373–13602	12373–13602	12402–13631

Table S2. The lengths and nucleotide composition of the entire mt genome, protein coding genes (PCG), RNA genes and tRNA genes of *Macropostrongyloides* species.

Taxon		Length (bp)	A (%)	T (%)	G (%)	C (%)	A+T (%)
M. baylisi	entire sequence	13,633	31.0	46.0	15.7	7.3	77
	PCG	10,255	28.6	47.3	16.5	7.6	75.9
	RNA genes	1,659	37.4	42.2	14.1	6.3	79.6
	tRNA genes	1,264	39.0	40	14.6	6.3	79
M. mawsonae	entire sequence	13,678	30.2	46.5	16.3	6.9	76.8
	PCG	10,259	27.6	48.1	17.1	7.2	75.6
	RNA genes	1,653	37.6	42.2	13.9	6.3	79.8
	tRNA genes	1,272	37.9	40.6	15.3	6.2	78.5
M. spearei	entire sequence	13,674	30.7	45.9	16.0	7.4	76.7
	PCG	10,260	28.3	47.1	16.8	7.9	75.4
	RNA genes	1,652	37.9	42.4	13.6	6.1	80.3
	tRNA genes	1,266	37.8	40.6	15.3	6.2	78.4
M. woodi	entire sequence	13,847	30.7	46.5	15.6	7.2	77.2
	PCG	10,255	28.1	47.7	16.6	7.6	75.8
	RNA genes	1,789	37.3	43.5	13	6.1	80.8
	tRNA genes	1,269	38.1	41.1	14.7	6.2	79.1
M. dissimilis	entire sequence	13,691	29.9	46.4	16.7	7	76.3
	PCG	10,262	27.5	47.8	17.5	7.2	75.3
	RNA genes	1,651	36.9	42.4	14.5	6.2	79.3

	tRNA genes	1,289	36.5	41.3	15.2	7	77.8
M. lasiorhini	entire sequence	13,720	29.9	46.5	16.5	7.2	76.4
	PCG	10,261	27.4	47.8	17.3	7.5	75.2
	RNA genes	1,656	36.8	42.2	14.7	6.3	79
	tRNA genes	1,279	37.9	41.1	14.5	6.4	79
M. yamagutii	entire sequence	13,686	30.6	45.6	16.6	7.2	76.2
	PCG	10,263	27.9	47.2	17.5	7.5	72
	RNA	1,655	38.4	41.5	13.8	6.3	79.9
	tRNA	1,274	38	40.1	15.6	6.3	78.1

Table S3. The amino acid (aa) sequence lengths, initiation (ini.), and termination (ter.) codons of the mt protein coding genes (PCG) of *Macropostrongyloides* species.

PCG	*M. baylisi*		*M. mawsonae*		*M. spearei*		*M. woodi*		*M. dissimilis*		*M. lasiorhini*		*M. yamagutii*	
	aa.	ini. /ter.	aa.	ini. /ter.	aa.	ini. /ter.	aa.	ini. /ter.	aa.	ini. /ter.	aa.	ini. /ter.	aa.	ini. /ter.
*cox*1	524	ATA/TAA	524	ATA/TAA	524	ATA/TAA	524	ATA/TAA	524	ATA/TAA	524	ATA/TAA	524	ATA/TAA
*cox*2	232	ATA/TAA	232	ATT/TAA	232	ATT/TAA	232	ATA/TAA	232	ATT/TAA	232	ATT/TAA	232	ATA/TAA
*nad*3	112	TTG/TAA	112	TTG/TAG	112	TTG/TAA	112	ATA/TAG	112	ATT/TAG	112	ATA/TAG	112	ATA/TAA
*nad*5	520	ATA/T*	520	ATA/T*	521	ATA/TAG	521	ATA/TAG	520	ATG/T*	520	ATA/T*	520	ATG/T*
*nad*6	145	ATA/TAA	145	ATT/TAA	145	GTG/TAA	145	ATT/TAA	145	ATC/TAG	145	ATA/TAA	145	TTG/TAA
*nad*4L	78	ATT/TAA	78	ATT/TAA	78	ATT/TAA	78	ATT/TAA	78	ATT/TAA	78	ATT/TAG	78	ATT/TAG
*nad*1	285	ATA/TAG	285	ATA/TAG	285	ATA/TAA	285	ATA/TAA	285	ATA/TAG	285	ATA/TAG	285	ATA/TAA
*atp*6	200	ATT/TAA	200	ATT/TAA	200	ATT/TAA	200	ATT/TAA	200	ATT/TAA	200	ATT/TAA	200	ATT/TAA
*nad*2	280	ATA/TAG	281	ATA/TAG	281	ATG/TAG	279	ATA/TAG	281	ATG/TAA	280	ATT/TAG	281	ATG/TAA
cob	371	ATT/TAG	371	ATT/TAA	371	ATT/TAG	371	ATT/TAA	371	ATT/TAG	371	ATT/TAA	371	ATT/TAA
*cox*3	255	ATT/T*	255	ATA/T*	255	ATT/T*	255	ATA/T*	255	ATT/T*	255	ATA/T*	255	ATT/T*
*nad*4	410	TTG/TAA	410	TTG/TAA	410	TTG/TAA	410	TTG/TAA	410	TTG/TAG	410	TTG/TAA	410	TTG/TAA

*incomplete stop codons

Table S4. Codon usage in the 12 protein coding genes of *Macropostrongyloides* species shown as the number of occurrences of each codon followed by percentage of the total usage in parentheses.

Amino acid	Codon	21V1	W449	23Q1	23M1	10W9	41R1	DD4
**Non-polar**								
Alanine	GCG	13 (0.38)	9 (0.26)	7 (0.21)	8 (0.23)	4 (0.12)	8 (0.23)	7 (0.21)
	GCA	23 (0.67)	19 (0.56)	26 (0.76)	20 (0.59)	20 (0.59)	14 (0.41)	15 (0.44)
	GCT	62 (1.81)	70 (2.05)	65 (1.90)	67 (1.96)	44 (1.29)	39 (1.14)	39 (1.14)
	GCC	10 (0.29)	6 (0.18)	9 (0.26)	10 (0.29)	5 (0.15)	2 (0.06)	7 (0.21)
Isoleucine	ATT	261 (7.17)	251 (7.34)	255 (7.46)	265 (7.75)	257 (7.51)	210 (6.14)	262 (7.67)
	ATC	8 (0.06)	2 (0.06)	16 (0.47)	11 (0.32)	13 (0.38)	14 (0.41)	18 (0.53)
Leucine (UUR)	TTG	144 (4.21)	166 (4.86)	161 (4.71)	140 (4.10)	137 (4.01)	137 (4.01)	129 (3.78)
	TTA	354 (10.36)	326 (9.53)	321 (9.39)	363 (10.62)	266 (7.78)	304 (8.89)	244 (7.14)
Leucine (CUN)	CTG	3 (0.09)	4 (0.12)	8 (0.23)	2 (0.06)	11 (0.32)	27 (0.80)	9 (0.26)
	CTA	8 (0.23)	11 (0.32)	16 (0.47)	11 (0.32)	17 (0.5)	47 (1.38)	7 (0.21)
	CTT	17 (0.5)	18 (0.53)	18 (0.53)	11 (0.32)	35 (1.02)	51 (1.49)	15 (0.44)
	CTC	1 (0.03)	2 (0.06)	1 (0.03)	0 (0)	4 (0.12)	7 (0.21)	6 (0.18)
Methionine	ATG	50 (1.46)	63 (1.84)	60 (1.75)	65 (1.90)	69 (2.02)	69 (2.02)	49 (1.43)
	ATA	193 (5.65)	187 (5.47)	189 (5.53)	185 (5.41)	115 (3.36)	151 (4.42)	111 (3.25)
Phenylalanine	TTT	430 (12.58)	429 (12.54)	423 (12.37)	428 (12.52)	424 (12.4)	411 (12.00)	441 (12.8)
	TTC	5 (0.15)	6 (0.18)	12 (0.35)	10 (0.29)	38 (1.11)	36 (1.05)	42 (1.23)
Proline	CCG	9 (0.26)	6 (0.18)	4 (0.12)	6 (0.18)	3 (0.09)	5 (0.15)	1 (0.03)
	CCA	16 (0.47)	16 (0.47)	27 (0.79)	21 (0.61)	16 (0.47)	15 (0.44)	9 (0.26)
	CCT	47 (1.38)	53 (1.55)	42 (1.23)	45 (1.32)	38 (1.11)	31 (0.91)	33 (0.97)
	CCC	7 (0.21)	5 (0.15)	7 (0.21)	8 (0.23)	4 (0.12)	7 (0.21)	9 (0.26)
Tryptophan	TGG	22 (0.64)	25 (0.73)	23 (0.67)	26 (0.76)	44 (1.29)	51 (1.49)	41 (1.20)
	TGA	45 (1.32)	42 (1.23)	44 (1.29)	41 (1.20)	61 (1.78)	36 (1.05)	47 (1.37)
Valine	GTG	30 (0.88)	49 (1.43)	39 (1.14)	34 (0.10)	42 (1.23)	46 (1.34)	31 (0.91)
	GTA	98 (2.87)	87 (2.54)	98 (2.87)	88 (2.58)	64 (1.87)	89 (2.60)	69 (2.02)
	GTT	112 (3.28)	127 (3.71)	99 (2.90)	111 (3.25)	133 (3.89)	106 (3.10)	110 (3.22)
	GTC	8 (0.23)	2 (0.06)	9 (0.26)	7 (0.21)	14 (0.41)	9 (0.26)	11 (0.32)
**Polar**								
Aspargine	AAT	134 (3.92)	138 (4.03)	139 (4.06)	132 (3.86)	150 (4.39)	98 (2.87)	176 (5.15)
	AAC	10 (0.29)	4 (0.12)	3 (0.09	8 (0.23)	11 (0.32)	9 (0.26)	21 (0.61)

Cysteine	TGT	40 (1.17)	39 (1.14)	44 (1.29)	41 (1.20)	62 (1.81)	76 (2.22)	81 (2.37)
	TGC	2 (0.059)	3 (0.09)	1 (0.03)	2 (0.06)	17 (0.50)	7 (0.21)	18 (0.53)
Glutamine	GAG	15 (0.44)	30 (0.88)	12 (0.35)	29 (0.85)	40 (1.17)	18 (0.53)	29 (0.85)
	GAA	27 (0.79)	45 (1.36)	30 (0.88)	46 (1.35)	36 (1.01)	25 (0.73)	30 (0.88)
Glycine	GGG	48 (1.40)	24 (0.70)	49 (1.43)	46 (1.35)	42 (1.23)	44 (1.29)	46 (1.35)
	GGA	43 (1.26)	49 (1.43)	35 (1.02)	39 (1.14)	39 (1.14)	25 (0.73)	45 (1.32)
	GGT	90 (2.63)	116 (3.39)	102 (2.98)	104 (3.04)	87 (2.54)	93 (2.72)	85 (2.49)
	GGC	7 (0.21)	6 (0.16)	7 (0.21)	6 (0.18)	6 (0.18)	5 (0.15)	18 (0.53)
Serine (UCN)	TCG	10 (0.29)	6 (0.16)	10 (0.29)	7 (0.21)	10 (0.29)	9 (2.63)	7 (0.21)
	TCA	55 (1.61)	49 (1.43)	49 (1.43)	50 (1.46)	52 (1.52)	26 (0.76)	53 (1.55)
	TCT	68 (1.99)	83 (2.43)	76 (2.22)	79 (2.31)	46 (1.35)	53 (1.55)	50 (1.46)
	TCC	6 (0.18)	2 (0.06)	6 (0.18)	9 (0.26)	4 (0.12)	7 (0.21)	14 (0.41)
Serine (AGN)	AGG	34 (1.00)	49 (1.43)	45 (1.32)	41 (1.20)	42 (1.23)	36 (1.05)	62 (1.81)
	AGA	89 (2.60)	72 (2.11)	78 (2.28)	63 (1.84)	47 (1.37)	55 (1.61)	64 (1.87)
	AGT	97 (2.83)	100 (2.93)	92 (2.69)	110 (3.22)	94 (2.75)	88 (2.57)	94 (2.75)
	AGC	10 (0.29)	3 (0.09)	13 (0.38)	8 (0.23)	5 (0.15)	4 (0.12)	13 (0.38)
Threonine	ACG	12 (0.35)	14 (0.40)	9 (0.26)	11 (0.32)	5 (0.15)	13 (0.38)	13 (0.38)
	ACA	31 (0.90)	27 (0.79)	37 (1.08)	33 (0.97)	25 (0.73)	16 (0.47)	27 (0.79)
	ACT	74 (2.17)	73 (2.13)	68 (1.99)	71 (2.08)	53 (1.55)	47 (1.38)	50 (1.46)
	ACC	2 (0.059)	5 (0.15)	3 (0.09)	2 (0.06)	10 (0.29)	4 (0.12)	14 (0.41)
Tyrosine	TAT	177 (5.18)	187 (5.47)	180 (5.26)	169 (4.94)	195 (5.70)	213 (623)	211 (6.17)
	TAC	15 (0.44)	4 (0.12)	7 (0.21)	13 (0.38)	18 (0.53)	27 (0.79)	34 (0.99)
**Acidic**								
Aspartate	GAT	56 (1.64)	59 (1.73)	58 (1.70)	61 (1.79)	66 (1.93)	62 (1.81)	64 (1.87)
	GAC	5 (0.15)	3 (0.09)	5 (0.15)	1 (0.03)	7 (0.20)	6 (0.18)	6 (0.18)
Glutamate	GAG	30 (0.88)	30 (0.88)	23 (0.67)	29 (0.85)	40 (1.17)	33 (0.97)	29 (0.85)
	GAA	47 (1.38)	45 (1.32)	52 (1.52)	46 (13.46)	36 (1.05)	49 (1.43)	30 (0.88)
**Basic**								
Arginine	CGG	1 (0.03)	2 (0.06)	0 (0.00)	3 (0.09)	5 (0.15)	7 (0.21)	3 (0.09)
	CGA	2 (0.06)	2 (0.06)	3 (0.09)	3 (0.09)	1 (0.03)	4 (0.12)	4 (0.12)
	CGT	27 (0.79)	26 (0.76)	27 (0.79)	25 (0.73)	15 (0.44)	23 (0.67)	21 (0.62)
	CGC	1 (0.03)	1 (0.03)	1 (0.03)	0 (0)	0 (0)	2 (0.06)	2 (0.06)
Histidine	CAT	49 (1.43)	52 (1.52)	49 (1.43)	50 (1.46)	58 (1.67)	41 (1.20)	28 (0.82)

	CAC	6 (0.18)	3 (0.09)	8 (0.23)	6 (0.18)	2 (0.06)	9 (0.26)	4 (0.12)
Lysine	AAG	27 (0.79)	28 (0.82)	20 (0.59)	30 (0.88)	49 (1.43)	33 (0.97)	52 (1.52)
	AAA	85 (2.49)	82 (2.40)	89 (2.60)	83 (2.43)	77 (2.25)	73 (2.14)	71 (2.08)

Table S5. Summary of the raw data generated from the next-generation sequencing of mitochondrial genomes of *Macropostrongyloides* species.

Species	Voucher ID	Total reads (mil)	Coverage*	No. of reads mapped to mt genome
M. baylisi	21V1	1.4	88	16,438
M. dissimilis	10W9	2.7	19	18,026
M. lasiorhini	41R1	5.4	17	16,261
M. mawsonae	W449	1.9	211	69,481
M. spearei	23Q1	2.8	617	239,184
M. woodi	23M1	3.4	71	30,048
M. yamagutii	DD4	13	14	12,916

*Coverage C = (L*Ck)/(L-k+1), where: L=read length, Ck=kmer coverage, k=Spades kmer size (Spades kmer default length = 21)

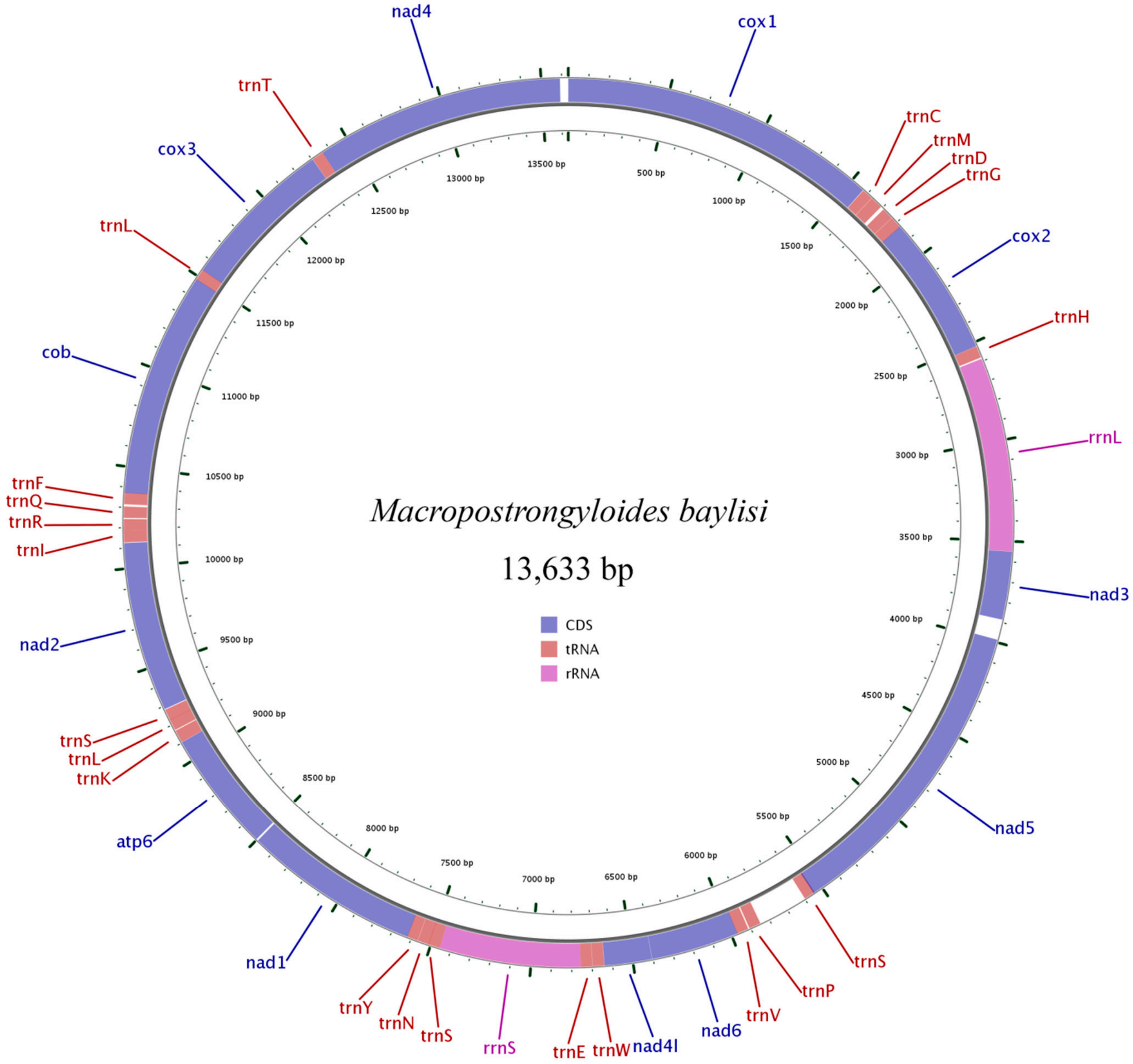

Figure S1. Schematic representation of the mitochondrial genome of *Macropostrongyloides* represented by the genome of *M. baylisi*. The protein coding sequences (CDS) are shown in blue, the transfer RNAs (tRNA) are shown in red, the large (rrnL) and small (rrnS) ribosomal RNAs are shown in pink.
